# Experimental signatures of the transition from acoustic plasmon to electronic sound in graphene

**DOI:** 10.1126/sciadv.adi0415

**Published:** 2023-09-29

**Authors:** David Barcons Ruiz, Niels C.H. Hesp, Hanan Herzig Sheinfux, Carlos Ramos Marimón, Curdin Martin Maissen, Alessandro Principi, Reza Asgari, Takashi Taniguchi, Kenji Watanabe, Marco Polini, Rainer Hillenbrand, Iacopo Torre, Frank H.L. Koppens

**Affiliations:** ^1^ ICFO - Institut de Ciències Fotòniques, The Barcelona Institute of Science and Technology, Av. Carl Friedrich Gauss 3, 08860 Castelldefels (Barcelona), Spain.; ^2^ CIC nanoGUNE, 20018 Donostia-San Sebastián, Spain.; ^3^ Department of Physics and Astronomy, The University of Manchester, M13 9PL Manchester, UK.; ^4^ School of Physics, Institute for Research in Fundamental Sciences, IPM, Tehran 19395-5531, Iran.; ^5^ School of Physics, University of New South Wales, Kensington, NSW 2052, Australia.; ^6^ International Center for Materials Nanoarchitectonics, National Institute for Materials Science, 1-1 Namiki, Tsukuba 305-0044, Japan.; ^7^ Research Center for Functional Materials, National Institute for Materials Science, 1-1 Namiki, Tsukuba 305-0044, Japan.; ^8^ Dipartimento di Fisica, Università di Pisa, Largo Bruno Pontecorvo 3, I-56127 Pisa, Italy.; ^9^ Istituto Italiano di Tecnologia, Graphene Labs, Via Morego 30, I-16163 Genova, Italy.; ^10^ CIC nanoGUNE BRTA and Department of Electricity and Electronics, UPV/EHU, 20018 Donostia-San Sebastián, Spain.; ^11^ IKERBASQUE, Basque Foundation for Science, Bilbao, Spain.; ^12^ ICREA-Institucio Catalana de Recerca i Estudis Avancats, 08010 Barcelona, Spain.

## Abstract

Fermi liquids respond differently to perturbations depending on whether their frequency is higher (collisionless regime) or lower (hydrodynamic regime) than the interparticle collision rate. This results in a different phase velocity between the collisionless zero sound and the hydrodynamic first sound. We performed terahertz photocurrent nanoscopy measurements on graphene devices, with a metallic gate close to the graphene layer, to probe the dispersion of propagating acoustic plasmons, the counterpart of sound modes in electronic Fermi liquids. We report the observation of a change in the plasmon phase velocity when the excitation frequency approaches the electron-electron collision rate that is compatible with the transition between the zero and the first sound mode.

## Teaser

The transition from the zero to first sound in graphene is probed by imaging acoustic plasmons with THz photocurrent nanoscopy.

## INTRODUCTION

The Fermi liquid paradigm (*1*, *2*) is one of the cornerstones of modern condensed matter theory, providing an effective description of the many-body systems whose elementary excitations are weakly interacting fermionic quasi-particles. The theory of Fermi liquids provides an understanding of why conduction electrons in metals behave essentially as noninteracting particles.

Fermi liquids can support collective modes in the form of longitudinal density oscillations that are analogous to sound in classical fluids. Their propagation depends on whether the angular frequency ω of the mode is higher or lower than the interparticle collision rate (*3*) $\tau_{\mathrm{coll}}^{-1}$. Liquid ^3^He, a neutral Fermi liquid, was the first system in which the transition (a change in the phase velocity and attenuation of the propagating mode) from the first sound mode ($\omega \ll \tau_{\mathrm{coll}}^{-1}$, i.e., in the hydrodynamic regime) to the zero sound mode ($\omega \gg \tau_{\mathrm{coll}}^{-1}$, i.e., in the collisionless regime) was observed (*4*).

In electronic Fermi liquids with long-range Coulomb interactions, where the electron-electron (ee) scattering time τ_ee_ plays the role of τ_coll_, first and zero sound collapse into a plasmon mode (*5*). In such a mode, the smooth crossover from the collisionless to the hydrodynamic regime manifests in the dispersion relation ω(*q*) only at subleading order in the wave vector *q* of the mode (*5*), and it is therefore very challenging to observe. However, two-dimensional (2D) electron liquids allow for sufficient screening of the long-range part of the Coulomb interaction by a nearby metallic gate electrode, the first sound and zero sound reappear (*5*–*7*), and a transition between the two can be observed. In 2D electron liquids with screened ee interactions, the zero sound mode is known as acoustic plasmon and has been extensively studied experimentally in hexagonal boron nitride (hBN)–encapsulated graphene devices (*8*, *9*). However, to the best of our knowledge, the electronic first sound mode, the closest electronic analog of ordinary sound, has never been observed experimentally.

**Fig. 1.**
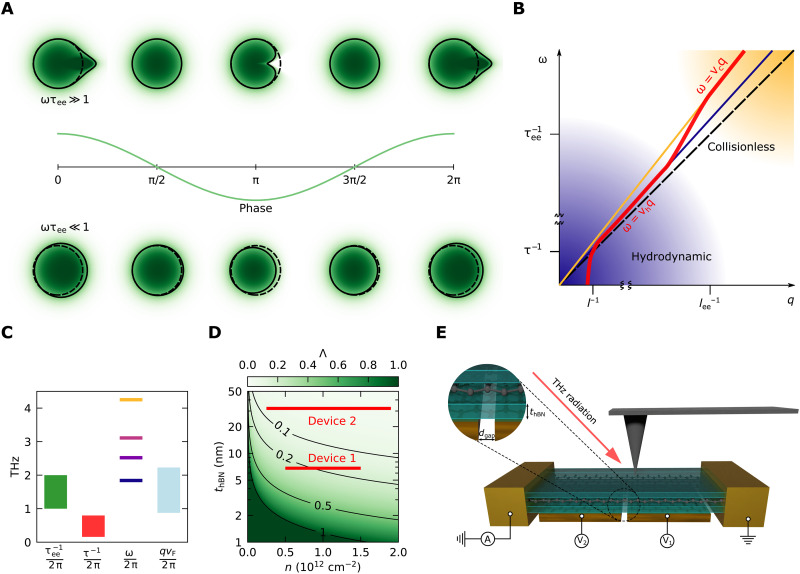
Electronic sound transition in graphene. (**A**) Fermi surface deformation associated with an acoustic plasmon propagating along the positive *x* direction in the collisionless (top row) and hydrodynamic (bottom row) regime. The green shading represents the electron’s distribution function, and the black line represents the set of points where the distribution function is 1/2. The dashed line marks the position of the equilibrium Fermi sphere. The oscillation represents the potential density perturbation. The phase is given by *qx* − ω*t*. See also movies S1 and S2 for an animation of the Fermi surface deformation. (**B**) Qualitative sketch of the dispersion of acoustic plasmons (thick red line) highlighting the change in phase velocity between the two regimes. The yellow and blue lines represent the phase velocity in the collisionless and hydrodynamic regimes, respectively. The black dashed line marks the Fermi velocity. (**C**) Frequency scales involved in our experiment. The laser frequency ω can be tuned to be larger or smaller than the ee collision rate $\tau_{\mathrm{ee}}^{-1}$, while the momentum relaxation rate τ^−1^ is always the slowest mechanism. *qv_F_* is always smaller than ω because the plasmon phase velocity never reaches the Fermi velocity *v_F_* for the values of the screening parameter Λ in our experiment. $\tau_{\mathrm{ee}}^{-1}$ has been calculated for our devices’ parameters (note S7), while τ^−1^ has been extracted from the high-frequency data (note S5). (**D**) Screening parameter Λ for single-layer graphene as a function of the hBN thickness *t*_hBN_ and carrier density. Red lines represent the data ranges for our devices once the air gap is taken into account (note S3). (**E**) Schematic view of the dual-gated device geometry and THz nanoscopy experiment. The inset highlights the relevant geometric dimensions.

In this work, we report a change in the phase velocity of acoustic plasmons that is compatible with the transition between an acoustic plasmon and the electronic first sound. We probe this transition at room temperature (RT) using a terahertz (THz) source whose angular frequency ω can be tuned around the ee collision rate $\tau_{\mathrm{ee}}^{-1}$, with τ_ee_ being 0.1 to 0.2 ps in doped graphene (*10*–*12*). Figure 1A and movies S1 and S2 show the evolution of the distribution function of electrons during the propagation of a plasmon mode. While for $\omega \gg \tau_{\mathrm{ee}}^{-1}$ the distribution function differs significantly from the equilibrium one, for $\omega \ll \tau_{\mathrm{ee}}^{-1}$ ee, collisions have time to smooth the distribution to a circle, leading to a quasi-equilibrium, fluid-like response (*13*). This results in a change in the phase velocity *v*_p_ of the mode between the two regimes (see Fig. 1B) from the collisionless value *v*_c_ to the hydrodynamic value *v*_h_.

The plasmon velocity difference has, at long wavelength, an intuitive physical interpretation thanks to an analogy with viscoelastic materials (*14*). Materials respond as solids (with an elastic shear force) to shear deformations that are faster than a certain equilibration time scale τ_coll_ and as fluids (with a dissipative shear force) to shear deformations slower than τ_coll_. This time scale greatly varies (even by orders of magnitude) depending on the material and diverges for ordered solids. Electrons are no exception to this behavior, τ_coll_ = τ_ee_. In the collisionless regime, the elastic shear force, which is not present in the hydrodynamic regime, adds to the Coulomb and pressure forces (*14*) in sustaining the plasmon mode. This makes the plasmon mode stiffer, increasing its velocity. To observe this effect, it is, however, necessary to screen the otherwise dominant Coulomb force.

The electronic first sound pertains to the hydrodynamic regime, which is characterized by τ_ee_ being the shortest time scale of the system (*15*–*17*). Quantitatively, this happens when τ_ee_ ≪ τ, ω^−1^, (*qv*_F_)^−1^, where τ is the momentum relaxation time, ω^−1^ is the time over which the phase of the mode changes significantly, and (*qv*_F_)^−1^ (*q* being the wave vector of the mode) is the time it takes to an electron traveling at the Fermi velocity *v*_F_ to cross a substantial fraction of a spatial oscillation of the mode (the corresponding ranges of frequencies relevant for our experiment are depicted in Fig. 1C). Because both $\tau_{\mathrm{ee}}^{-1}$ and τ^−1^ increase with temperature, typically at different rates, the hydrodynamic regime can only be realized in high-mobility electronic systems for a limited window of experimental conditions (*10*, *18*, *19*). The hydrodynamic regime has been demonstrated experimentally in encapsulated graphene samples (*10*, *11*, *20*–*23*) or GaAs/AlGaAs quantum wells (*19*, *24*).

The transition between the zero sound and the first sound in 2D electronic liquid was studied theoretically (*5*–*7*) using simplified models based on the semiclassical Boltzmann transport equation that captures nonlocal effects (*9*, *25*). The magnitude of the difference between the plasmon velocity in the two regimes is controlled by the screening parameter (*7*)

$$\text{Λ}=\frac{C}{e^{2}N}\approx \frac{1}{t_{\mathrm{hBN}}[\mathrm{nm}]\sqrt{|n[10^{12}\cdot cm^{-2}]|}}$$ (1)

where *C* is the capacitance per unit area between the electron liquid and the metallic gate, *e* is the unit charge, and *N* is the electronic density of states at the chemical potential. The definition of Λ used here differs slightly from the one used in (*7*) in that the density of states *N* appearing in Eq. 1 is the observed or renormalized one, not the bare one. The second relation holds for the specific case of single-layer graphene with carrier density *n* and separated from a nearby metallic gate by an hBN spacer of thickness *t*_hBN_. Figure 1D shows the values of Λ that can be reached as a function of the experimental parameters.

The sought effect is negligible for Λ ≈ 0 but becomes strong for Λ ≈ 1. When the latter condition is reached, the hydrodynamic plasmon velocity becomes even smaller than *v*_F_. In the extreme case of very large screening (Λ ≫ 1), the collisionless plasmon velocity tends to *v*_F_, while the hydrodynamic plasmon velocity tends to the 2D energy-wave (second sound) velocity $v_{F}/\sqrt{2}$ (*26*). The convergence of these two modes to the same limiting velocity can be understood because they both approach charge-neutral oscillations. In the case of the second sound, this happens because of the charge compensation between electrons and holes, while in the case of acoustic plasmons, the same happens because of the compensation due to induced image charges in the metallic gate.

On the basis of the theoretical model presented in (*5*–*7*) and making an approximation that is well justified in single-layer graphene (*27*) (i.e., neglecting the first-order spin-symmetric Landau parameter $F_{1}^{s}$ that controls the many-body renormalization of the Drude weight), it is possible to derive (note S4) a simple relation between the collisionless plasmon velocity *v*_c_ and the hydrodynamic plasmon velocity *v*_h_

$$v_{h}=\sqrt{\frac{v_{c}^{2}+v_{c}\sqrt{v_{c}^{2}-v_{F}^{2}}}{2}}$$ (2)

with *v*_F_ as the (renormalized) Fermi velocity. From this formula, we immediately see that the difference between the two velocities is negligible if *v*_c_ ≫ *v*_F_ (corresponding to small values of Λ) and becomes most important when *v*_c_ ≈ *v*_F_. Even in this extreme case, the relative difference between the two velocities cannot exceed ∣*v*_h_ − *v*_c_∣/*v*_c_ ≲ 29%.

## RESULTS

In this work, we probe the transition between the collisionless and the hydrodynamic regime of electrons in graphene by measuring the phase velocity of acoustic plasmons at different angular frequencies close to the expected value of $\tau_{\mathrm{ee}}^{-1}$ that we calculated for the specific structures of our experiment (see Fig. 1C and note S7). To this aim, we fabricated two hBN-encapsulated single-layer graphene devices, dubbed device 1 and device 2, respectively, with different gate-graphene separations. The transition effect is observed in device 1, while device 2 is a control device in which this effect is predicted to be negligible. The two devices share the same split-gate configuration depicted in Fig. 1E. They consist of hBN/graphene/hBN heterostructures on top of metallic palladium gates. Each metallic gate is split into two halves whose voltages can be controlled separately. This allows the creation of a sharp p-n junction in the sample, which enables the thermoelectric detection of the plasmonic field (*28*).

The only relevant difference between the two devices is the thickness of the bottom hBN spacer (t_hBN_ in Fig. 1E) that is chosen to be as small as possible (t_hBN_ = 2.0 nm, leading to a design value of Λ ≈ 0.5 at the carrier densities used in the experiment) in device 1 and larger (t_hBN_ = 11.8 nm, corresponding to Λ ≈ 0.08) in device 2. This means that the Coulomb interaction should be strongly screened in device 1 (quantified by higher values of Λ as seen in Fig. 1D), where acoustic plasmons are expected to propagate with low velocity (with *v*_p_/*v*_F_ reaching values as low as 1.5). This yields a sizable change in *v*_p_ between the two regimes. On the contrary, in device 2, the Coulomb interaction is less screened (see Fig. 1D) and *v*_p_/*v*_F_ never goes below 2.5. This means that, in device 2, *v*_p_ is almost the same in the two regimes, and no significant transition effect is expected.

**Fig. 2.**
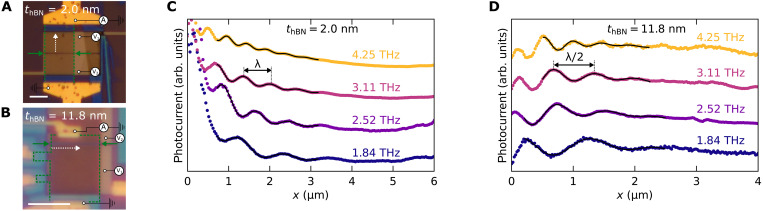
Photocurrent nanoscopy of acoustic graphene plasmons. (**A**) Optical micrographs of device 1, indicating the electrodes used for collecting the photocurrent signal and the gate electrodes. The white dashed line marks where datasets in (C) were acquired. The green dashed lines delimitate the area covered by graphene, and the green arrows the location of the junction. Scale bars, 5 μm. (**B**) Same as (A) for device 2, here, the white dashed line marks where datasets in (D) were acquired. (**C**) Near-field photocurrent signal acquired in device 1 along the line shown in (A) at four different frequencies. The carrier density is fixed at *n* ≈ 10^12^ cm^−2^. In this configuration (perpendicular to the junction), only λ-fringes appear. Data are shifted vertically for more clarity. (**D**) Same as in (C) for device 2, along the line marked in (B). In this configuration (parallel to the junction), only λ/2-fringes appear. The color code is the same as in (C).

We performed THz photocurrent nanoscopy (*8*) measurements at RT (*T* = 295 K) in a commercial scanning near-field optical microscope (SNOM). We used a methanol gas laser to measure at four different frequencies (*f* = 1.84, 2.52, 3.11, 4.25 THz). For each laser frequency, we scanned the tip repeatedly along the white dashed lines indicated in Fig. 2 (A and B), for a set of gate voltages *V*_1_, while the other gate is kept at a voltage *V*_2_ chosen to maximize the photocurrent signal (see Materials and Methods). The dominating plasmon launching mechanism differs between the two devices due to the very different vertical confinement of the plasmon [see (*9*) and note S1]. In device 1, the sharp metallic edge at the junction launches a plane wave propagating perpendicular to it. By scanning the tip perpendicular to the junction, we measure λ-fringes (i.e., fringes with a periodicity equal to the plasmon wavelength). In device 2, the tip launches a circular plasmonic wave. By scanning the tip parallel to the junction, we detect λ/2-fringes (i.e., fringes with a periodicity equal to half the plasmon wavelength) due to the standing wave originating from the plasmons reflected at the graphene edge and traveling back to the tip (*29*). Instead, we do not observe plasmonic oscillations in device 1 when scanning parallel to the junction, nor in device 2 when scanning perpendicular to the junction. In both cases, the SNOM tip serves as a local probe to rectify the plasmonic field, generating heat, which is then converted into photocurrent at the p-n junction via thermoelectric effect (*28*).

Figure 2 (C and D) displays the real part of the photocurrent signal (recorded at the first harmonic of the tip frequency) acquired for both devices at the four studied frequencies, at a carrier density *n* ≈ 10^12^ cm^−2^. Both devices, and particularly device 1, display very high electronic quality with plasmon lifetimes of about 0.5 to 1 ps (note S5). This is evident from the quality of the data in Fig. 2 (C and D), which show up to 7 clearly visible oscillation fringes. The presence of a good number of fringes is pivotal for the reliable extraction of the plasmon wavelength. Because of the different scanning directions, in device 1, there is an additional decay of the signal along the scanning direction as the tip moves away from the junction (*28*). Conversely, in device 2, the tip-junction distance is kept constant, but there is a geometric decay due to the cylindrical plasmonic wave radiating away from the tip (notes S1 and S2). The high signal-to-noise ratio typical of our technique and the high mobility of our devices allows us to accurately extract the plasmon wavelength λ_p_ = 2π/*q*_p_ for carrier densities above 0.5 × 10^12^ cm^−2^ for device 1 and above 0.3 × 10^12^ cm^−2^ for device 2 (see note S2 for the full data sets and the fitting procedure details).

**Fig. 3.**
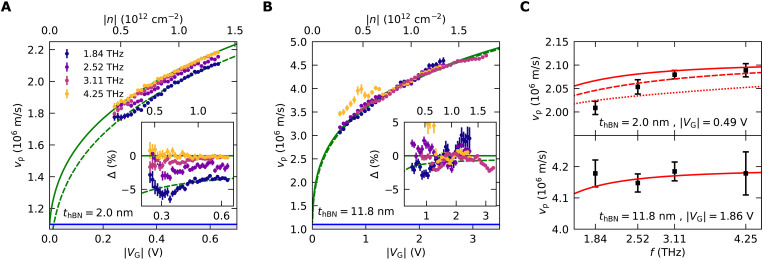
Frequency dependence of the velocity of acoustic graphene plasmons. (**A**) Measured plasmon phase velocity as a function of the gate voltage with respect to the Dirac point ∣*V*_G_∣ and carrier density for device 1 for the frequencies indicated in the legend. The solid green line is a one-parameter fit *v*_c_(*V*_G_) of the data at 4.25 THz. The green dashed line is the corresponding hydrodynamic velocity obtained by applying Eq. 2 to *v*_c_(*V*_G_). The inset shows the relative variation of the plasmon velocity with respect to the fit, Δ = *v*_p_(*V*_G_, ω)/*v*_c_(*V*_G_) − 1, as a function of the gate voltage. The blue-shaded region indicates the area below the Fermi velocity. (**B**) Same as in (A) for the control device 2. (**C**) Plasmon phase velocity as a function of the frequency for device 1 (top) and device 2 (bottom) for a *V*_G_ corresponding to a carrier density of *n* ≈ 10^12^ cm^−2^. The data points and error bars have been obtained by fitting the dispersions in (A) and (B) to the functional form explained in the text. The solid red lines follow the expected plasmon velocity for our devices’ parameters (see table S1) using the model in (*7*). The dashed and dotted red lines correspond to a value of τ_ee_ (0.16 ps for device 1 and 0.17 ps for device 2) reduced by a factor of 2 and 5, respectively. The difference between the three lines is not visible for device 2.

From these measured values of λ_p_, we extract the plasmon phase velocity as a function of the gate voltage (Fig. 3, A and B), measured with respect to the charge neutrality point, determined by two-probe transconductance measurements, *V*_G_ = *V*_1_ − *V*_1,CNP_, for device 1 and 2, respectively. We were able to extract the plasmon wavelength with a sufficient degree of accuracy at the four laser frequencies. Measuring at lower laser frequencies was not possible because λ_p_ becomes too large compared with device dimensions, propagation, and cooling length, making the extraction of the wavelength not reliable enough. From the measured plasmon velocity, we find (see note S3) a smaller capacitance than the one expected from the thickness of the exfoliated hBN flakes in both devices, yielding typical values of Λ of ≈0.2 and ≈0.04 in devices 1 and 2, respectively. We attribute this discrepancy to air gaps between the metallic gate and the hBN flake (see note S3). This effect can be described as an effective hBN thickness larger than the nominal one. We used this more realistic quantity to locate our devices in the parameter space plotted in Fig. 1D.

## DISCUSSION

The most notable observation is that the two devices show a different frequency dependence of the plasmon velocity. In contrast to device 2 (Fig. 3B), the data of device 1 (Fig. 3A) show a clear frequency dependence, with the plasmon velocity slowing down by ≈5% from the highest frequency to the lowest. The effect is emphasized in the inset that shows the relative variation with respect to the highest frequency. We will show that our findings are compatible with the transition from the collisionless to the hydrodynamic regime. To this aim, we compare Eq. 2 with our data by approximating the collisionless velocity *v*_c_ with our velocity data at the highest available frequency, 4.25 THz, and the Fermi velocity with its value calculated at the carrier density of *n* = 10^12^ cm^−2^, *v*_F_ ≃ 1.1 × 10^6^ m/s (note S3). For more clarity, instead of applying Eq. 2 directly, we fit the experimental data with a simplified one-parameter (α) model derived from the collisionless expression by removing quantum capacitance and many-body renormalization effects (note S4) $v_{c}(V_{G})=v_{F}\;(\alpha \sqrt{|V_{G}|}+1){\left(2\alpha \sqrt{|V_{G}|}+1\right)}^{-1/2}$(green solid line in Fig. 3A). The parameter α, related to the capacitance quantifies the impact of gating on the plasmonic dispersion. We then apply Eq. 2 to the fitted curve to obtain our theoretical estimate of the hydrodynamic velocity (green dashed line in Fig. 3A). The theoretical line matches well with the data extracted at the lowest available frequency of 1.84 THz. As expected, repeating the same procedure with the control device, we find only a very small velocity shift that is compatible with experimental errors, as shown in Fig. 3B. In Fig. 3C, we display the plasmon velocity for a fixed carrier density *n* ≈ 10^12^ cm^−2^ as a function of the laser frequency. To reduce the uncertainty associated with the use of a single experimental point in voltage, the data points and their error bars have been obtained by fitting the experimental plasmon dispersions in Fig. 3 (A and B) to a functional expression, which allows fitting the dispersion in both the hydrodynamic and collisionless regime: *v*_p_(*V*_G_) = *a*∣*V*_G_∣^1/4^(1 + *b*∣*V*_G_∣). The rationale behind this expression is that for small screening and neglecting all quantum and many-body effect, the plasmon velocity is proportional to ∣*V*_G_∣^1/4^, while all the effects beyond this approximation can be captured in a small voltage interval by a linear factor (1 + *b*∣*V*_G_∣).

We note that (*5*–*7*) also predict a change in the plasmon damping rate Γ ≈ ω Re{*q*_p_}/Im{*q*_p_} at the crossover between the hydrodynamic and collisionless regime. However, while the quality of our results allows a reliable extraction of Re{*q*_p_}, the extraction of Im{*q*_p_} has a larger uncertainty. On top of that, the plasmon decay due to Im{*q*_p_} needs to be disentangled (in the case of device 1) from decay due to the varying tip-junction distance. As a result, we do not aim to observe the transition in the damping rate measurements (note S2).

Motivated by the good matching between the expected hydrodynamic velocity and the experimental data at 1.84 THz, we aim to make a thorough comparison between our experiment and the theoretical model in (*7*). This model needs, as input, the value of Λ, τ, τ_ee_, *v*_F_, and of the zeroth-order Landau parameter (*1*) [which is related to the compressibility correction $F_{0}^{s}$ (*9*)] and allows the calculation of the plasmon wave vector at every frequency. Our best estimates of these parameters, either measured or calculated using the theory presented in (*30*, *31*), are summarized in table S1, with details on how they are obtained given in notes S3 and S5 to S7. The calculated plasmon velocity according to these parameters is shown as a solid line in Fig. 3C for the two devices. While the trend is correct, the model predicts that only a smaller shift should be observed in our experimental range. We attribute this discrepancy mainly to an overestimate of τ_ee_ in our many-body calculation (τ_ee_ = 0.16 ps for device 1 and τ_ee_ = 0.17 ps for device 2) that pushes the central frequency of the transition *f*_tr_ = (2πτ_ee_)^−1^ to around 1 THz, below our experimental points. To support this notion, we show in Fig. 3C the theoretical line calculated with the same parameters but with τ_ee_ reduced by a factor of 2, which makes *f*_tr_ fall inside our experimental range (dashed line), and factor of 5, which makes *f*_tr_ above the highest measured frequency (dotted line). This shows that a better agreement is reached by assuming that the real value of τ_ee_ is reduced by around a factor of 2 with respect to the calculated one. This discrepancy could motivate future theoretical investigations.

Further mismatch between the theoretical prediction and the experiment can be attributed to the estimate of other parameters or to mechanisms that are not captured by the simplified theoretical model in (*5*–*7*). In particular, each angular harmonic of the one-particle distribution function relaxes, in principle, according to a different scattering rate (*32*, *33*). In addition, considering effects beyond the energy-independent relaxation time approximation may be important for a quantitative description of the transition.

The dispersive behavior of the dielectric environment could also introduce a frequency dependence in our experiment. However, the hBN permittivity change in our frequency range is too small to explain the effect (note S8), and the palladium gate electrode has perfect mirror response in the same range (its plasma frequency being close to 10 eV) (*34*).

In conclusion, we have experimentally demonstrated a shift in the phase velocity of acoustic graphene plasmons in a graphene sample with a very thin (2 nm) hBN spacer when the frequency is tuned from 4.25 to 1.84 THz. The same effect was not observed in a device with a thicker hBN spacer. The magnitude of the observed shift and the frequency at which the shift is happening are in qualitative agreement with the theoretical expectation for the collisionless to hydrodynamic transition.

Two main ingredients have allowed us to observe the shift in the phase velocity. First, we have produced high-mobility graphene devices in which the fastest scattering event is ee collisions. Second, we have incorporated a metallic gate electrode in very close proximity to the graphene sheet to ensure sufficient screening of the long-range Coulomb interaction, achieving record-low values of acoustic graphene plasmon velocity. Our results can stimulate further experimental investigation on the dynamical aspects of the hydrodynamic regime of electronic transport.

The ee collision rate strongly depends on temperature (*10*). Performing experiments in a variable-temperature cryogenic near-field microscope (*35*) would permit studying the evolution of the hydrodynamic regime as a function of temperature. Moreover, the hydrodynamic regime could be studied by using THz graphene plasmon cavities coupled with a continuously tunable THz source in the few THz range. Last, interesting nonlinear plasmonic effects are predicted to happen in the hydrodynamic regime due to the nonlinearities of the Navier-Stokes equations in graphene (*36*, *37*).

A recently published paper (*38*) reports the observation of hydrodynamic plasmons and energy waves in graphene using on-chip THz spectroscopy. We believe that the findings of this complementary work strengthen the validity and importance of our results.

## MATERIALS AND METHODS

### Device fabrication

We start with the fabrication of the metallic gates. We use standard electron beam lithography (EBL) at 30 kV to define two rectangles separated by 200- to 300-nm gaps on a 270-nm-thick polymethyl methacrylate layer. After developing, we perform a plasma descum at low power to remove resist leftovers. We deposit 2 nm of Ti and 15 nm of Pd, both by electron beam evaporation. Last, to remove the spikes at the edges of the gates, we anneal the samples at 300°C in Ar/H_2_ for 3 hours. We check with atomic force microscopy (AFM) and choose only the gates without spikes. We find that the gap is in the order of 100 to 200 nm.

We mechanically exfoliate hBN and graphene flakes on SiO2/Si chips and carefully choose the desired hBN flakes for our devices. To assemble the hBN/graphene/hBN heterostructure, we use polycarbonate stamps and drop the heterostructure onto the pre-patterned metallic gates at 160°C (*39*). Last, we define the edge contacts (*40*) and shape the graphene channel with EBL and reactive ion etching.

Before our near-field measurements, we clean the surface of the samples with an AFM tip in contact mode (*41*), applying forces between 30 and 60 nN. In fig. S1, we show an AFM image of the surface of device 2 after the AFM cleaning.

### THz photocurrent nanoscopy measurements

As the laser source, we used two far-infrared gas lasers: FIRL-100 (Edinburgh Instruments Ltd.) and SIFIR-50 (Coherent Inc.). Both lasers output the same THz lines at very similar powers.

As the nearfield microscope, we used a neaSNOM (neaspec GmbH). Because we perform photocurrent measurements, we removed the interferometer to maximize the power at the tip. The photocurrent signal (typically in the order of few nA) is read out through a photocurrent amplifier (DHPCA-100 from FEMTO Messtechnik GmbH), working at gains between 10^4^ and 10^6^ V/A, depending on the device resistance and laser power. The amplifier output is fed to the neaSNOM lock-in input, such that the collected signal is demodulated at the harmonics of the tip frequency. We used an Au-coated AFM tip with 250-nm radius at the apex and a force constant of 3 N/m, model LRCH250 (Team Nanotec GmbH). The photocurrent signal is demodulated at either the first or second harmonic of the tip frequency (~75 KHz), and the first harmonic of the mechanical phase is subtracted (*9*, *28*). The typical tapping amplitude is 80 to 120 nm.

First, we locate a clean line perpendicular to the junction for device 1 and parallel to it but close enough to maximize the signal for device 2. Whether we want to measure on the electron or hole doping regimes, we choose a different gate voltage for the other gate electrode to maximize the photocurrent. We scan along the same line for a range of gate voltages. In fig. S2, we display the raw measurements acquired for device 1 (left) and device 2 (right), where in the horizontal axis we scan the tip, and in the vertical axis we step the gate voltage.

We check for position and carrier density drifts between scans that may alter the data. We always scan across the p-n junction (device 1) or across the graphene edge (device 2). This, together with comparing forward and backward traces (which are recorded sequentially), allows us to discard sample drifts that could lead to an apparent change in the fringe spacing. To check for carrier density drifts, i.e., a drift of the charge neutrality point *V*_CNP_, we verify that the gate voltage at which the photocurrent signal changes its sign remains the same. Moreover, we do not expect this to happen in samples with a local gate. This samples are much less affected by drift and hysteresis than encapsulated samples directly on top of SiO_2_ due to the lack of dielectric-dielectric interfaces that may trap charges for long times.

## Supplementary Materials

### This PDF file includes:

Materials and Methods Notes S1 to S8 Figs. S1 to S11 Table S1 Legends for movies S1 and S2 References
