# Supplementary material for: Experimental signatures of the transition from acoustic plasmon to electronic sound in graphene
Source: Sci Adv. 2023 Sep 29;9(39):eadi0415. doi: 10.1126/sciadv.adi0415 (PMC10541005; doi:10.1126/sciadv.adi0415)
Supplement: Supplementary file 1 — Materials and Methods Notes S1 to S8 Figs. S1 to S11 Table S1 Legends for movies S1 and S2 References [file sciadv.adi0415_sm.pdf]

Science Advances
AAAS

Supplementary Materials for

Experimental signatures of the transition from acoustic plasmon to electronic sound in graphene

David Barcons Ruiz *et al.*

Corresponding author: Frank H.L. Koppens, frank.koppens@icfo.eu; Iacopo Torre, iacopo.torre@icfo.eu

Sci. Adv. **9**, eadi0415 (2023)
DOI: 10.1126/sciadv.adi0415

The PDF file includes:

Materials and Methods
Notes S1 to S8
Figs. S1 to S11
Table S1
Legends for movies S1 and S2
References

Other Supplementary Material for this manuscript includes the following:

Movies S1 and S2

Supplementary Text

1. Plasmon launching: tip or junction

In our experiment, we can distinguish two types of plasmon launching in our devices (Fig. S3): tip-launched plasmons, which appear as λ/2-fringes, and junction-launched plasmons, appearing as λ-fringes in the experiment.

- In the case of tip-launched plasmons (λ/2-fringes), the tip provides the momentum mismatch to launch the plasmon in all directions (circular wave). Once it reaches the lithographically defined graphene edge, it is reflected and travels back to the tip.
- In the case of junction-launched plasmons (λ-fringes), the launching happens at the sharp edge of the metallic gate. A sharp metallic edge can compensate the momentum mismatch between the incoming light and the plasmon-polariton, similar to what the SNOM tip does in most experiments. Due to the large length of the junction compared to the plasmon wavelength, we consider the plasmon as a plane wave propagating perpendicular to the junction.

2. Plasmon fringe fitting

As mentioned in the main text and in the Supplementary Note 1, we have two different plasmon launching mechanisms for device 1 and 2. Therefore, the fringe fitting strategy of the plasmon oscillations slightly differs.

- In device 1 (t_{hBN} = 2.0 nm), we observe only junction-launched plasmons (λ-fringes). The tip, which is scanned perpendicular to the junction, acts as a rectifier, thus converting the plasmon into heat (*28*). The heat is dissipated through the hBN (*42*) and in the graphene layer, rising the electronic temperature at the junction side where we rectify the plasmon. Since the Seebeck coefficient is tuned to different values at both sides of the junction, the temperature difference results in a thermo-voltage generation. The voltage difference generates a current flow which is the signal we measure. In summary, we need to consider λ-fringes which propagate over a characteristic distance l_p, being carried by a signal that decays nearly exponentially away from the junction, with a characteristic decay length l_T (cooling length). The latter should also be included as an extra decay channel for the oscillations as we scan away from the junction. We define $k_p^\dagger$ as the complex wavevector of the measured fringes. This is related to the plasmon wavevector by $\mathrm{Re}\{k_p^\dagger\} = \mathrm{Re}\{k_p\} = 2\pi/\lambda_p$ and $\mathrm{Im}\{k_p^\dagger\} \approx 1/l_p + 1/l_T$, where the second term accounts for heat loss related to the cooling length. The fitting function used read as

$$s(x) = \mathrm{Re}\left[(A + iB)e^{ik_p^\dagger x}\right] + Ce^{-x/l_T} + D \qquad (S1)$$

  where *A*, *B*, *C*, and *D* are real-valued amplitude coefficients.
- In device 2 (t_{hBN} = 11.8 nm), we did only observe λ/2-fringes (tip-launched plasmons) for the frequencies 1.84, 2.52 and 3.11 THz. However, at 4.25 THz we did observe both λ/2-fringes and λ-fringes. The tip is scanned parallel to the junction and acts as a rectifier the same way it does for device 1, but here we do not expect any decay of the signal due to the cooling length because of the fixed tip-junction distance. Close to the graphene edge, the

photocurrent signal decreases because of edge effects and geometrical effects due to the position of the contacts. This variation close to the edge is taken into account by subtracting a smooth background signal from the fits.

At the three lowest frequencies (1.84, 2.52 and 3.11 THz) we fit the photocurrent signal with the following function:

$$s(x) = s_{bg}(x) + \mathrm{Re}\left[(A + iB)e^{i2k_\mathrm{p}x}\right] \qquad (S2a)$$

where $\mathrm{Im}\{k_\mathrm{p}\} = 2\pi/l_\mathrm{p}$, A and B are real-valued amplitude coefficients, and the background signal $s_{bg}(x)$ is a fourth order polynomial.

Instead, at 4.25 THz we need to include λ-fringes as well to obtain an accurate value of the decay for the plasmon and carrier lifetime analysis (Supplementary Note 5). Only for this frequency, we use the following function :

$$s(x) = s_{bg}(x) + \frac{A}{\sqrt{x}}\cos\left(2\mathrm{Re}\{k_\mathrm{p}\}x + \varphi\right)e^{-2\mathrm{Im}\{k_\mathrm{p}\}x} + \frac{B}{x}\cos\left(\mathrm{Re}\{k_\mathrm{p}\}x + \theta\right)e^{-\mathrm{Im}\{k_\mathrm{p}\}x} \quad (S2b)$$

where A and B are real-valued amplitude coefficients, φ and θ angles to account for the global and relative phase of the two oscillations, and $s_{bg}(x)$ is a third order polynomial.

In both cases the fringe fitting was performed excluding the first oscillation that is too distorted by edge effects. For device 1, the imaginary part of the plasmon wavevector is affected by a larger error due to the subtraction of two contributions. In Fig. S4 we show $\mathrm{Re}\{k_\mathrm{p}\}$, and $\mathrm{Im}\{k_\mathrm{p}\}$ (for device 1 we plot $\mathrm{Im}\{k_\mathrm{p}^\dagger\}$), extracted from the fits, as a function of frequency and gate voltage V_1.

3. <u>Capacitance and carrier density determination</u>

To convert the gate voltage V_1 to carrier density according to $n = -eC_\mathrm{tot}\,(V_1 - V_{1,\mathrm{CNP}})$, we include in the total capacitance the capacitance of an hBN layer with a known thickness t_hBN from AFM, an air gap t_air between the metallic gate and the hBN due to possible contamination or surface roughness, and the quantum capacitance $C_q(n)$ of graphene. This gives

$$\frac{1}{C_{tot}} = \frac{1}{C} + \frac{1}{C_q} = \frac{1}{\frac{\epsilon_0\epsilon_\mathrm{hBN}}{t_\mathrm{hBN}}} + \frac{1}{\frac{\epsilon_0}{t_\mathrm{air}}} + \frac{1}{\frac{e^2}{2}DoS(n)}\,, \qquad (S3)$$

where ϵ_0 is the vacuum permittivity, ϵ_hBN is the out of plane dielectric constant of hBN ($\epsilon_\mathrm{zz,hBN} = 3.56$) and $DoS(n) = 2\sqrt{|n|}/(\sqrt{\pi}\hbar v_\mathrm{F})$ is the density-dependent density of states in graphene.

With this relation and the parameters $\tau, \tau_{ee}, F_0, v_F$ estimated or calculated (See following Supplementary Notes) the relation between voltage and plasmon wavelength given in Ref. (*7*) reduces to a model with one fit parameter, namely the thickness of the air layer.

In order to find this value, we fit the plasmon dispersion at 4.25 THz to the experimental data. We find $t_\mathrm{air}^\mathrm{device\,1} \approx 1.46$ nm and $t_\mathrm{air}^\mathrm{device\,2} \approx 6.24$ nm. This is in good agreement with the AFM imaging of the device, where we found $t_\mathrm{air}^\mathrm{device\,1} = (1.0 \pm 0.5)$ nm and $t_\mathrm{air}^\mathrm{device\,2} = (5.0 \pm 1.0)$ nm. The latter values present a large uncertainty due to the indirectness of the measurement. In fact, we first measured the thickness of the flakes before patterning the contacts and defining the channel. After the measurements, we measure the step height from the metallic gate to the top of the heterostructure (Fig. S5). Therefore, we could extract t_air from the measured step, $\Delta z = t_\mathrm{air} + t_\mathrm{hBN} + t_\mathrm{G} + t_\mathrm{top-hBN}$ and from the independent AFM measurements for hBN flake thicknesses $t_\mathrm{top-hBN}^\mathrm{device\,1} = 10.8$ nm and $t_\mathrm{top-hBN}^\mathrm{device\,2} = 15.0$ nm (we assume $t_\mathrm{G} = 0.3$ nm).

4. Plasmon phase velocity in the collisionless and hydrodynamic regime

The plasmon phase velocity in the collisionless and hydrodynamic regime are given in Eqs. (10-12) of Ref. (*7*). These two formulas drastically simplify if the Landau parameter of order one F_1^s is neglected. This is a good approximation in single-layer graphene since F_1^s is not constrained by Galileian invariance (allowing a many-body renormalization of the Drude weight) and the expected values of F_1^s are small (*7*). After making this approximation, expressing the many-body renormalizations in terms of Landau parameters, and adapting to the notations used in this work the two equations read

$$v_c = S_c = v_F \frac{1 + \Lambda^{-1} + F_0^s}{\sqrt{1 + 2\Lambda^{-1} + 2F_0^s}}, \qquad (S4)$$

$$v_h = S_h = v_F \sqrt{\frac{1 + \Lambda^{-1} + F_0^s}{2}}. \qquad (S5)$$

Here, F_0^s is the zero-order, spin-symmetric Landau parameter.
It must be noted that here v_F is the observed, i.e. renormalized Fermi velocity, corresponding to v_F^* in Ref. (*7*), moreover Λ is defined using the renormalized density of states and corresponds to the quantity $(v_F^*/v_F)\,\Lambda$ in the notation used in Ref. (*7*).
In our work we define the plasmon velocity as the phase velocity of the mode, i.e. $v_p(\omega) = \omega/Re[q_p(\omega)]$. Ref. (*7*) defines instead the plasmon velocity as $S_\omega = \omega/\sqrt{Re[q_p^2(\omega)]}$, the two definitions converging for small dissipation ($Im[q_p(\omega)] \ll Re[q_p(\omega)]$). In both the collisionless and hydrodynamic limit $\omega \gg \tau^{-1}$ and v_p coincides with S. We also checked that in all our datasets the difference between v_p and S is negligible thanks to the high quality of our samples.
Solving (S4) for the quantity $1 + \Lambda^{-1} + F_0^s$ and substituting back into (S5) yields the relation in Eq. (2) of the main text.

5. Plasmon and carriers lifetimes

For the highest frequency (4.25 THz) the imaginary part of the plasmon wavevector can be extracted. This allows to calculate the plasmon damping rate according to $\Gamma_\omega = (\omega Im[q_p^2\,(\omega)])/(2Re[q_p^2\,(\omega)])$ as in Ref. (*7*). The results are shown in Fig. S6, and yield plasmon lifetimes (inverse of the damping rate) of the order of 0.5-1 ps.
We note that this definition of the plasmon damping rate differs slightly from the one used in most of the plasmonic literature $\Gamma(\omega) = (\omega\, Im[q_p(\omega)])/(Re[q_p(\omega)])$. Again, the two definitions converge for $Im[q_p(\omega)] \ll Re[q_p(\omega)]$ and the difference is irrelevant in all our datasets.
The plasmon damping rates in the collisionless and hydrodynamic regime are given in Eq. (11-13) of Ref. (*7*). After making the same approximation and change of notations as discussed in the previous section we get

$$\Gamma_c = \tau^{-1} \frac{1 + \Lambda^{-1} + F_0^s}{1 + 2\Lambda^{-1} + 2F_0^s} + \tau_{ee}^{-1} \frac{1 + \Lambda^{-1} + F_0^s}{(1 + 2\Lambda^{-1} + 2F_0^s)^2}, \qquad (S6)$$

$$\Gamma_h = \frac{\tau^{-1}}{2} + \frac{\omega^2}{4\,(\tau^{-1} + \tau_{ee}^{-1})(1 + \Lambda^{-1} + F_0^s)}. \qquad (S7)$$

Where we remember that τ is the momentum relaxation scattering time i.e. the time that is extracted from mobility measurements. In the simple with low screening ($\Lambda \ll 1$) both formulas reduce to

$$\Gamma = \frac{\tau^{-1}}{2}\,. \qquad (S8)$$

To extract the momentum relaxation time τ we use the following procedure. We assume that at 4.25 THz the collisionless limit is fully developed and we can use formulas (S4-S6). At each voltage point we calculate the quantity $1 + \Lambda^{-1} + F_0^s$ from (S4) using the measured value of the plasmon velocity and a fixed value of $v_F = v_F(n = 10^{12}\ \mathrm{cm}^{-2}) = 1.1 \cdot 10^6\ m/s$. We then substitute in (S6), and using the measured value of Γ (Fig. S7) and the fixed value of $\tau_{ee} = \tau_{ee}(n = 10^{12}\ \mathrm{cm}^{-2}) = 0.16$ ps we can find τ. The results are shown in Fig. S7 together with the estimation resulting from the simple application of (S8) that offers a lower bound for τ. Both values are in the range of expected mobility scattering times for this type of structures and choosing one or the other does not alter our conclusions.

6. Many body renormalization corrections

The many-body renormalization of the velocity can be calculated in graphene from the real part of the electronic self-energy according to the theory developed in Ref. (*31*). In Fig. S8 we show the numerical results obtained taking into account the electrostatic screening in the precise geometry of our heterostructures, including the air gaps discussed in Supplementary Note 3. The density dependence is weak and almost no difference is observed between the two devices.
A calculation of the random-phase-approximation (RPA) ground-state energy of the graphene electron liquid allows extracting the renormalized compressibility as described in Ref. (*31*). This, together with the renormalization of the Fermi velocity allows to extract the spin-symmetric Landau parameter of order zero F_0^s that enters in the expressions for the velocity of the plasmon mode. In Fig. S9 we show the results of the numerical calculation for our device geometries.

7. Electron-electron scattering time

The electron-electron scattering time τ_{ee} can be extracted from a calculation of the imaginary part of the G_0W self-energy of electrons in graphene (*30*). In Fig. S10 we report the results of numerical calculations for our relevant electronic density range and room temperature $T = 295$ K, taking into account the realistic dielectric screening in the heterostructures, including the air gaps discussed in Supplementary Note 3.

8. hBN permittivity frequency dependence

To discard that the observed effects could be originated by the frequency dependent hBN dielectric functions, we plot in Fig. S11 its in-plane $\varepsilon_\parallel$ and out-of-plane $\varepsilon_\perp$ permittivities as a function of frequency following the model in Ref. (*43*).
In the frequency range of our experiment, i.e. from 1.84 THz to 4.25 THz, the relative change of the real part of the hBN permittivities is $\Delta\varepsilon_\parallel/\varepsilon_\parallel = 0.24$ % and $\Delta\varepsilon_\perp/\varepsilon_\perp = 0.52$ %.

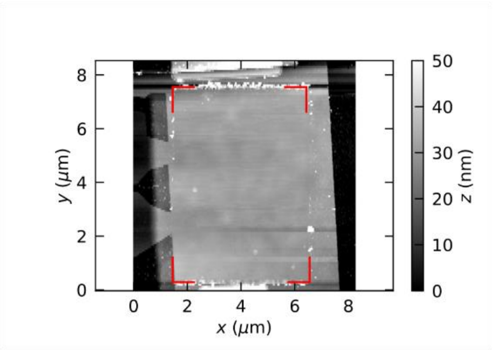

Fig. S1. AFM cleaning. AFM topography image of device 2 after AFM cleaning in contact mode the region inside the red marks.

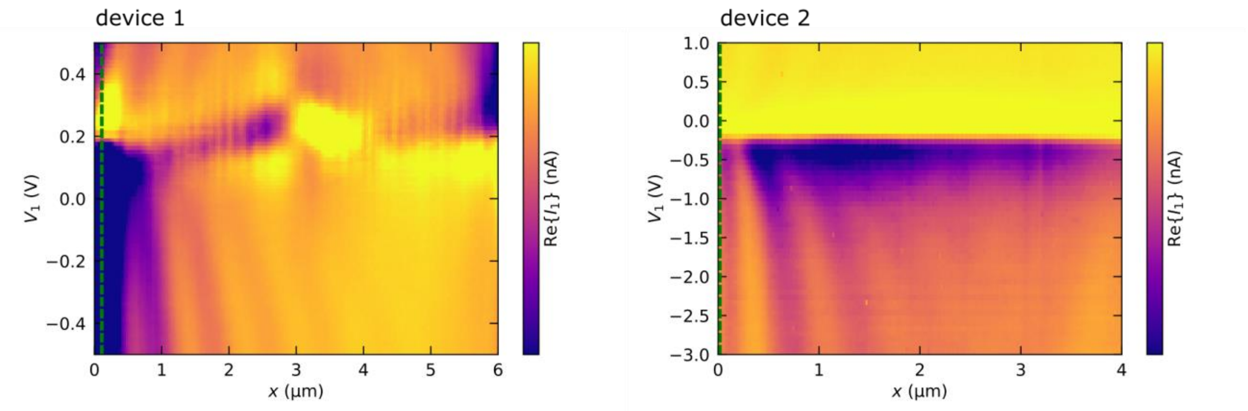

Fig. S2. Photocurrent nanoscopy maps. Photocurrent signal I_1 as a function of gate voltage V_1 and position *x*, for device 1 (left panel) and device 2 (right panel). The green dashed lines indicate the location of the junction and the graphene edge for device 1 and device 2, respectively. The laser frequency was 2.52 THz.

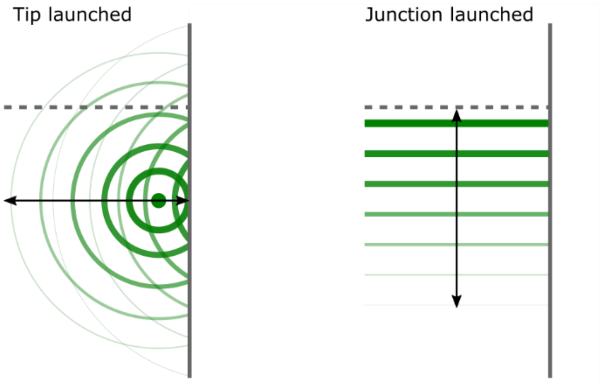

Fig. S3. Plasmon launching mechanisms. The green lines and circles indicate the plasmon wavefront. The black arrows indicate the scanning direction in the experiment. For tip-launched, it results in $\lambda/2$-fringes, while for junction-launched in λ-fringes. The solid grey line corresponds to the graphene edge and the dashed grey line to the location of the junction.

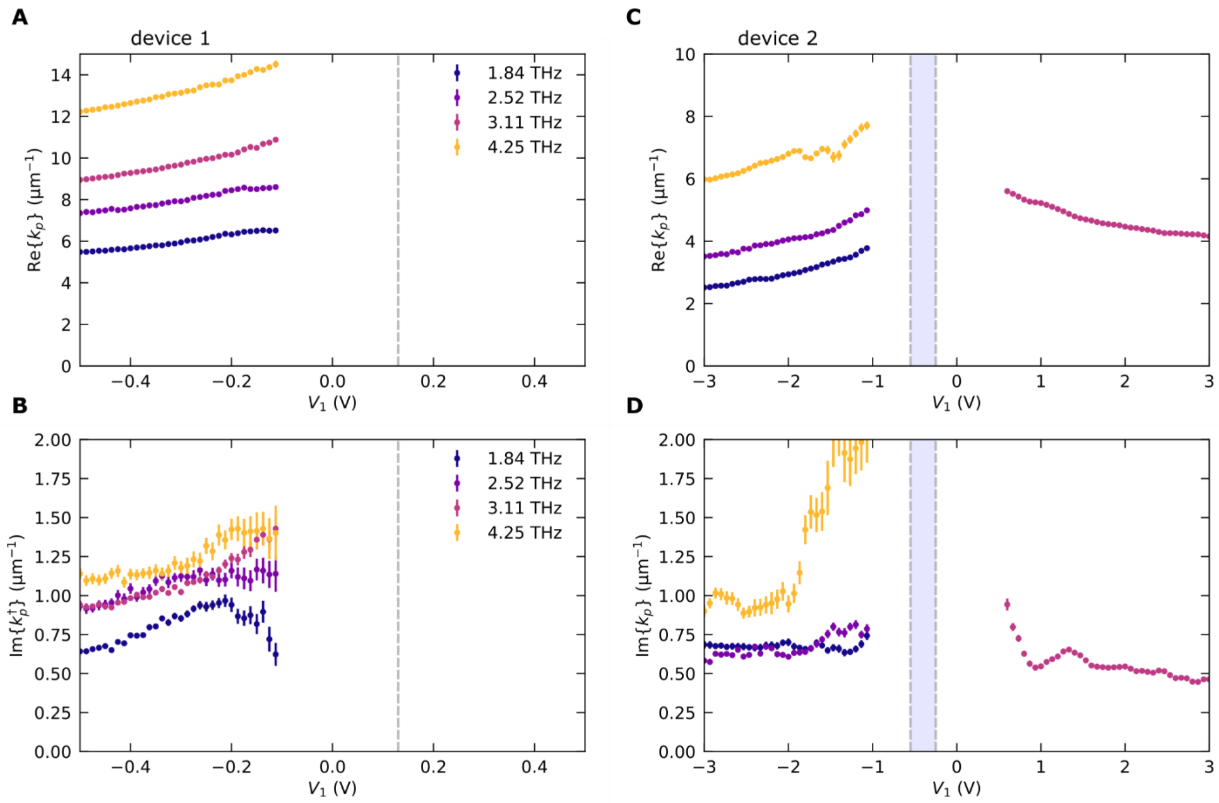

Fig. S4. Extracted real and imaginary part of the plasmon momentum. A,B. Extracted Re{k_p} and Im{$k_\mathrm{p}^\dagger$} as a function of gate voltage V_1, for device 1. **C,D.** Extracted Re{k_p} and Im{k_p} as a function of gate voltage V_1, for device 2. Vertical dashed lines indicate the position of charge neutrality point during the measurements, extracted from the two-terminal resistance. For device 2, there was a small shift during the measurements (considered in the analysis), indicated by the blue-shaded region.

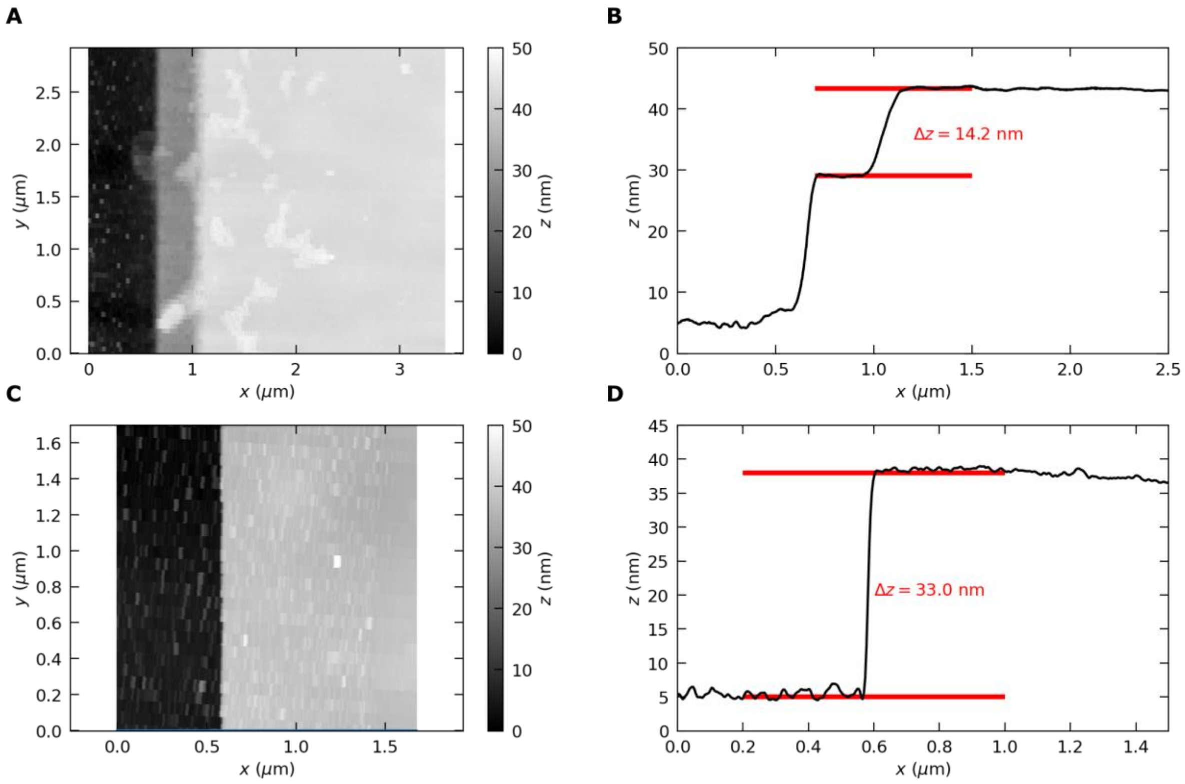

Fig. S5. **Heterostructure height measured with AFM. A.** AFM image of device 1 step from the metallic gate to the top of the hBN/graphene/hBN heterostructure. **B.** Step height profile extracted by averaging the AFM image in a. Δz indicates the total step height including the air gap discussed in the text. **C,D.** Same as **A,B** but for device 2, respectively.

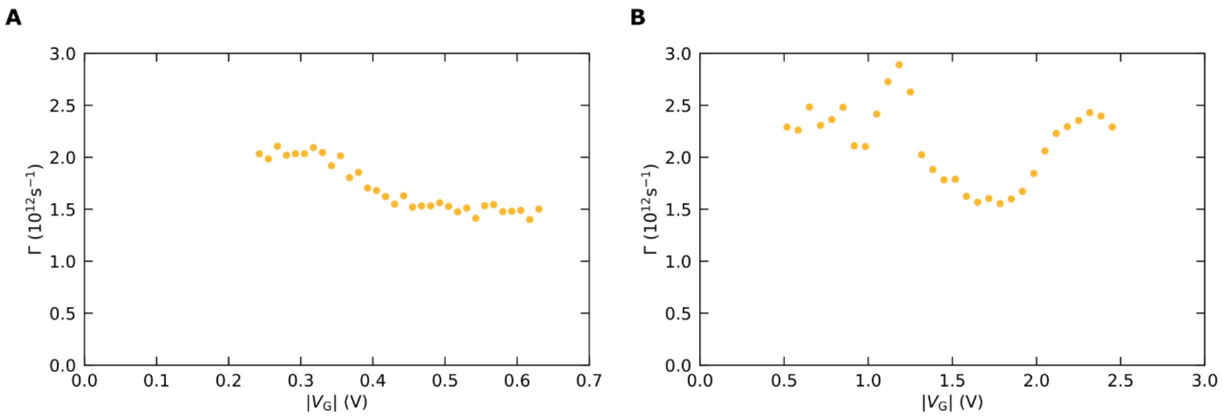

Fig. S6. Extracted plasmon damping. A. Plasmon damping rate Γ extracted from data at 4.25 THz in device 1. **B.** Same as in **A** for device 2.

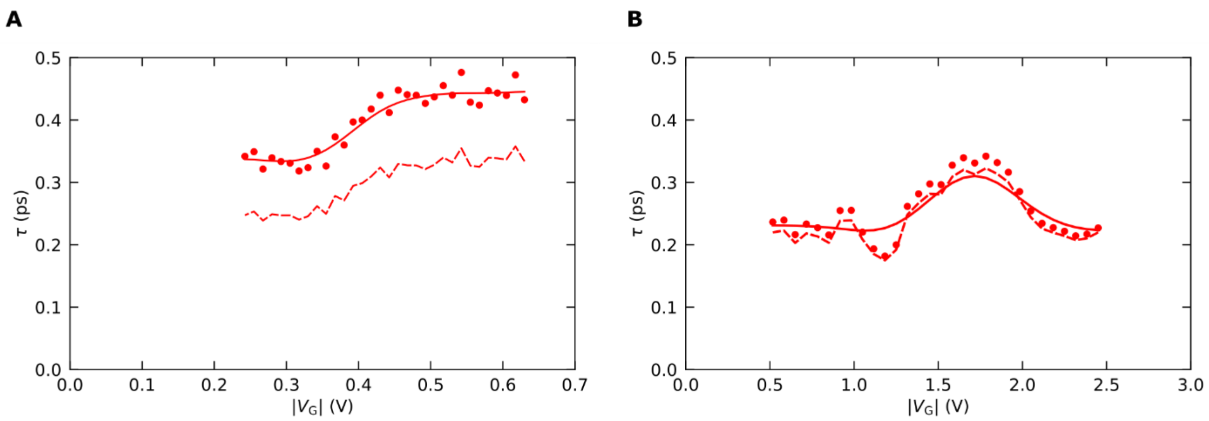

Fig. S7. Momentum-relaxing scattering time. A. Scattering time for momentum-relaxing collisions as a function of the gate voltage for device 1. Red dots are the result of the application of the procedure explained above, the dashed line results from the application of (S8), while the solid line is a smoothening of the experimental points. **B.** Same as in **A** for device 2.

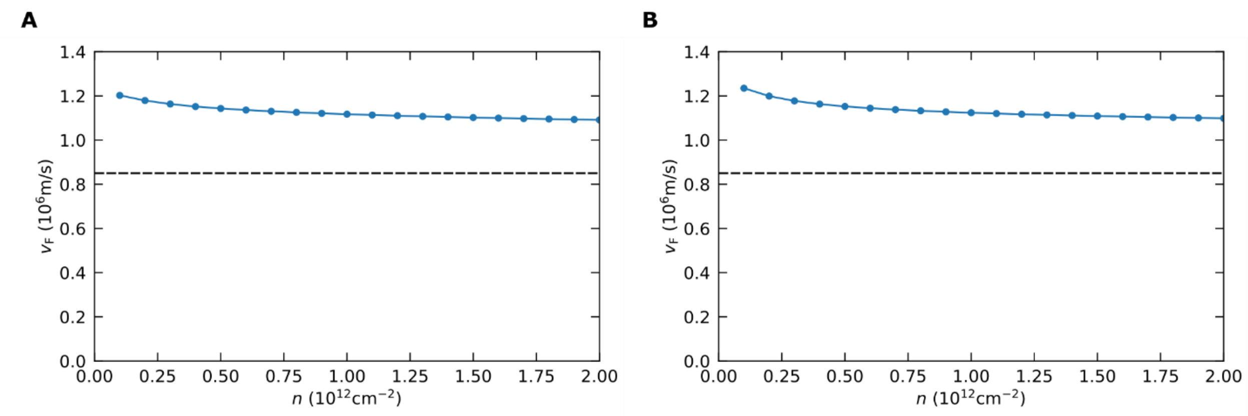

Fig. S8. Calculated Fermi velocity renormalization. A. Calculated many-body renormalized Fermi velocity in single-layer graphene, as a function of electronic density, taking into account the realistic dielectric environment of the heterostructure including the air gap for device 1. The black dashed line marks the bare value of the Fermi velocity $0.85 \cdot 10^6 \mathrm{m/s}$. **B.** Same as in **A** for device 2.

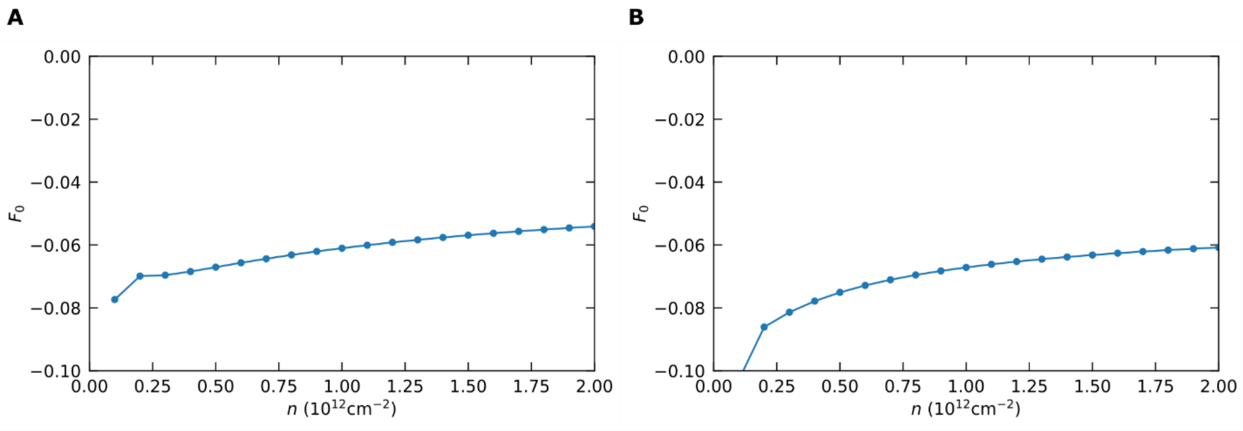

Fig. S9. Calculated Landau parameter F_0. A. Calculated spin-symmetric Landau-Fermi liquid parameter F_0 for single-layer graphene, as a function of electronic density, taking into account the realistic dielectric environment of the heterostructure including the air gap. **B.** Same as in **A** for device 2.

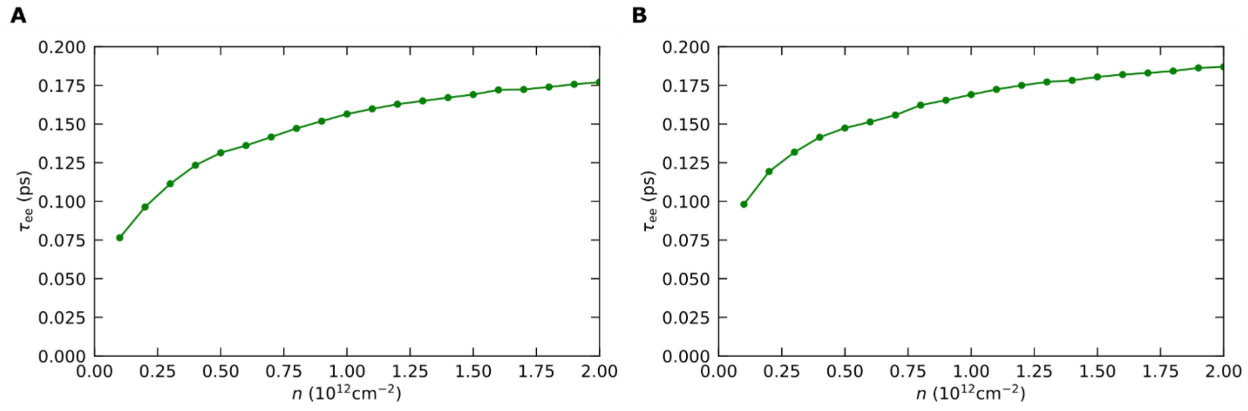

Fig. S10. Calculated electron-electron scattering time. A. Calculated electron-electron scattering time for single-layer graphene at room temperature, as a function of electronic density, taking into account the realistic dielectric environment of the heterostructure including the air gap. **B.** Same as in **A** for device 2.

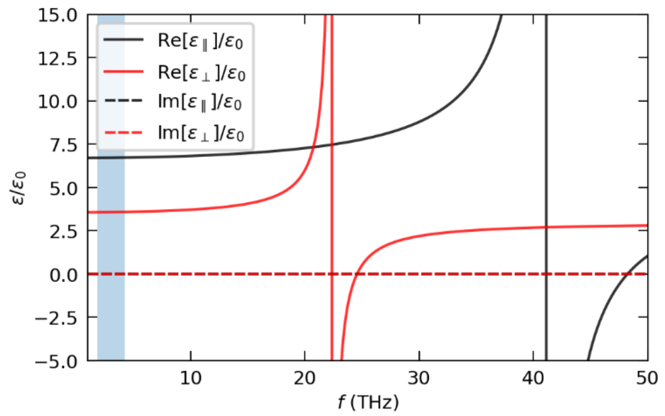

Fig. S11. hBN frequency-dependent permittivity. hBN permittivity as a function of frequency, based in the model in Ref. (*43*). All permittivities are normalized to the vacuum permittivity. Blue shaded region indicates the frequency range in our experiment.

Table S1. Summary of parameter used in Fig. 3C in the main text.

**Parameter**	**Device 1**	**Device 2**	**Source**
Air gap thickness t_{air}	1.46 nm	6.24 nm	Fitted (See Supp. Note 3)
Capacitance C	$4.37\ \mathrm{mF/m^2}$	$0.93\ \mathrm{mF/m^2}$	Extracted from t_{air} (See Supp Note 3)
Gate voltage V_G	0.49 V	1.86 V	Measured
Density n	$10^{12}\ \mathrm{cm^{-2}}$	$10^{12}\ \mathrm{cm^{-2}}$	Inferred from the two values above
Screening parameter Λ	0.178	0.038	Calculated with Eq. (1) with the renormalized Fermi Velocity
Fermi Velocity v_F	$1.1\ 10^6\ \mathrm{m/s}$	$1.1\ 10^6\ \mathrm{m/s}$	Calculated (See Supp. Note 6)
Zeroth-order Landau parameter F_0^s	-0.061	-0.067	Calculated (See Supp. Note 6)
Momentum relaxation time τ	0.44 ps	0.30 ps	Measured (See Supp. Note 5)
Electron-electron scattering time τ_{ee}	0.16 ps	0.17 ps	Calculated (See Supp. Note 7)

Video S1. Evolution of the Fermi surface distribution function in the collisionless regime as a function of phase (see Fig. 1A for more details).

Video S2. Evolution of the Fermi surface distribution function in the hydrodynamic regime as a function of phase (see Fig. 1A for more details).

REFERENCES AND NOTES

1. D. Pines, P. Nozières, *The Theory of Quantum Liquids* (W. A. Benjamin Inc. 1963).

2. R. Shankar, Renormalization-group approach to interacting fermions. *Rev. Mod. Phys.* **66**, 129–192 (1994).

3. I. M. Khalatnikov, A. A. Abrikosov, Dispersion of sound in a fermi liquid. *Soviet J. Exp. Theor. Phys.* **6**, 84–89 (1958).

4. W. R. Abel, A. C. Anderson, J. C. Wheatley, Propagation of zero sound in liquid He^3 at low temperatures. *Phys. Rev. Lett.* **17**, 74–78 (1966).

5. A. Lucas, S. Das Sarma, Electronic sound modes and plasmons in hydrodynamic two-dimensional metals. *Phys. Rev. B.* **97**, 115449 (2018).

6. D. Svintsov, Hydrodynamic-to-ballistic crossover in Dirac materials. *Phys.Rev. B.* **97**, 121405 (2018).

7. I. Torre, L. Vieira de Castro, B. Van Duppen, D. Barcons Ruiz, F. M. Peeters, F. H. L. Koppens, M. Polini, Acoustic plasmons at the crossover between the collisionless and hydrodynamic regimes in two-dimensional electron liquids. *Phys. Rev. B.* **99**, 144307 (2019).

8. P. Alonso-González, A. Y. Nikitin, Y. Gao, A. Woessner, M. B. Lundeberg, A. Principi, N. Forcellini, W. Yan, S. Vélez, A. J. Huber, K. Watanabe, T. Taniguchi, F. Casanova, L. E. Hueso, M. Polini, J. Hone, F. H. L. Koppens, R. Hillenbrand, Acoustic terahertz graphene plasmons revealed by photocurrent nanoscopy. *Nat. Nanotechnol.* **12**, 31–35 (2017).

9. M. B. Lundeberg, Y. Gao, R. Asgari, C. Tan, B. Van Duppen, M. Autore, P. Alonso-González, A. Woessner, K. Watanabe, T. Taniguchi, R. Hillenbrand, J. Hone, M. Polini, F. H. L. Koppens, Tuning quantum nonlocal effects in graphene plasmonics. *Science* **357**, 187–191 (2017).

10. D. A. Bandurin, I. Torre, R. K. Kumar, M. Ben Shalom, A. Tomadin, A. Principi, G. H. Auton, E. Khestanova, K. S. Novoselov, I. V. Grigorieva, L. A. Ponomarenko, A. K. Geim, M. Polini, Negative local resistance caused by viscous electron backflow in graphene. *Science* **351**, 1055–1058 (2016).

11. R. Krishna Kumar, D. A. Bandurin, F. M. D. Pellegrino, Y. Cao, A. Principi, H. Guo, G. H. Auton, M. Ben Shalom, L. A. Ponomarenko, G. Falkovich, K. Watanabe, T. Taniguchi, I. V. Grigorieva, L. S. Levitov, M. Polini, A. K. Geim, Superballistic flow of viscous electron fluid through graphene constrictions. *Nat. Phys.* **13**, 1182–1185 (2017).

12. D. A. Bandurin, A. V. Shytov, L. S. Levitov, R. K. Kumar, A. I. Berdyugin, M. Ben Shalom, I. V. Grigorieva, A. K. Geim, G. Falkovich, Fluidity onset in graphene. *Nat. Commun.* **9**, 4533 (2018).

13. L. P. Pitaevskiĭ, ZERO SOUND IN LIQUID He^3. *Sov. Phys. Usp.* **10**, 100–101 (1967).

14. S. Conti, G. Vignale, Elasticity of an electron liquid. *Phys. Rev. B.* **60**, 7966–7980 (1999).

15. R. N. Gurzhi, Hydrodynamic effects in solids at low temperature. *Sov. Phys. Usp.* **11**, 255–270 (1968).

16. I. Torre, A. Tomadin, A. K. Geim, M. Polini, Nonlocal transport and the hydrodynamic shear viscosity in graphene. *Phys. Rev. B.* **92**, 165433 (2015).

17. L. Levitov, G. Falkovich, Electron viscosity, current vortices and negative nonlocal resistance in graphene. *Nat. Phys.* **12**, 672–676 (2016).

18. D. Y. H. Ho, I. Yudhistira, N. Chakraborty, S. Adam, Theoretical determination of hydrodynamic window in monolayer and bilayer graphene from scattering rates. *Phys. Rev. B.* **97**, 121404 (2018).

19. M. J. M. de Jong, L. W. Molenkamp, Hydrodynamic electron flow in high-mobility wires. *Phys. Rev. B.* **51**, 13389–13402 (1995).

20. J. A. Sulpizio, L. Ella, A. Rozen, J. Birkbeck, D. J. Perello, D. Dutta, M. Ben-Shalom, T. Taniguchi, K. Watanabe, T. Holder, R. Queiroz, A. Principi, A. Stern, T. Scaffidi, A. K. Geim, S. Ilani, Visualizing Poiseuille flow of hydrodynamic electrons. *Nature* **576**, 75–79 (2019).

21. M. J. H. Ku, T. X. Zhou, Q. Li, Y. J. Shin, J. K. Shi, C. Burch, L. E. Anderson, A. T. Pierce, Y. Xie, A. Hamo, U. Vool, H. Zhang, F. Casola, T. Taniguchi, K. Watanabe, M. M. Fogler, P. Kim, A. Yacoby, R. L. Walsworth, Imaging viscous flow of the Dirac fluid in graphene. *Nature* **583**, 537–541 (2020).

22. P. Gallagher, C.-S. Yang, T. Lyu, F. Tian, R. Kou, H. Zhang, K. Watanabe, T. Taniguchi, F. Wang,

Quantum-critical conductivity of the Dirac fluid in graphene. *Science* **364**, 158–162 (2019).

23. A. Block, A. Principi, N. C. H. Hesp, A. W. Cummings, M. Liebel, K. Watanabe, T. Taniguchi, S. Roche, F. H. L. Koppens, N. F. van Hulst, K.-J. Tielrooij, Observation of giant and tunable thermal diffusivity of a Dirac fluid at room temperature. *Nat. Nanotechnol.* **16**, 1195–1200 (2021).

24. B. A. Braem, F. M. D. Pellegrino, A. Principi, M. Röösli, C. Gold, S. Hennel, J. V. Koski, M. Berl, W. Dietsche, W. Wegscheider, M. Polini, T. Ihn, K. Ensslin, Scanning gate microscopy in a viscous electron fluid. *Phys. Rev. B.* **98**, 241304 (2018).

25. D. Alcaraz Iranzo, S. Nanot, E. J. C. Dias, I. Epstein, C. Peng, D. K. Efetov, M. B. Lundeberg, R. Parret, J. Osmond, J.-Y. Hong, J. Kong, D. R. Englund, N. M. R. Peres, F. H. L. Koppens, Probing the ultimate plasmon confinement limits with a van der Waals heterostructure. *Science* **360**, 291–295 (2018).

26. T. V. Phan, J. C. W. Song, L. S. Levitov, Ballistic heat transfer and energy waves in an electron system. arXiv:1306.4972 [cond-mat.mes-hall] (2013).

27. S. H. Abedinpour, G. Vignale, A. Principi, M. Polini, W.-K. Tse, A. H. MacDonald, Drude weight, plasmon dispersion, and ac conductivity in doped graphene sheets. *Phys. Rev. B.* **84**, 45429 (2011).

28. M. B. Lundeberg, Y. Gao, A. Woessner, C. Tan, P. Alonso-González, K. Watanabe, T. Taniguchi, J. Hone, R. Hillenbrand, F. H. L. Koppens, Thermoelectric detection and imaging of propagating graphene plasmons. *Nat. Mater.* **16**, 204–207 (2017).

29. A. Woessner, M. B. Lundeberg, Y. Gao, A. Principi, P. Alonso-González, M. Carrega, K. Watanabe, T. Taniguchi, G. Vignale, M. Polini, J. Hone, R. Hillenbrand, F. H. L. Koppens, Highly confined low-loss plasmons in graphene–boron nitride heterostructures. *Nat. Mater.* **14**, 421–425 (2015).

30. A. Principi, G. Vignale, M. Carrega, M. Polini, Bulk and shear viscosities of the two-dimensional electron liquid in a doped graphene sheet. *Phys. Rev. B.* **93**, 125410 (2016).

31. R. Asgari, M. I. Katsnelson, M. Polini, Quantum capacitance and Landau parameters of massless Dirac fermions in graphene. *Ann. Phys.* **526**, 359–365 (2014).

32. P. J. Ledwith, H. Guo, L. Levitov, The hierarchy of excitation lifetimes in two-dimensional Fermi gases. *Ann. Phys. Rehabil. Med.* **411**, 167913 (2019).

33. J. Hofmann, S. Das Sarma, Collective modes in interacting two-dimensional tomographic Fermi liquids. *Phys. Rev. B.* **106**, 205412 (2022).

34. J. H. Weaver, Optical properties of Rh, Pd, Ir, and Pt. *Phys. Rev. B.* **11**, 1416–1425 (1975).

35. G. X. Ni, A. S. McLeod, Z. Sun, L. Wang, L. Xiong, K. W. Post, S. S. Sunku, B. Y. Jiang, J. Hone, C. R. Dean, M. M. Fogler, D. N. Basov, Fundamental limits to graphene plasmonics. *Nature* **557**, 530–533 (2018).

36. A. Tomadin, M. Polini, Theory of the plasma-wave photoresponse of a gated graphene sheet. *Phys. Rev. B.* **88**, 205426 (2013).

37. I. Torre, A. Tomadin, R. Krahne, V. Pellegrini, M. Polini, Electrical plasmon detection in graphene waveguides. *Phys. Rev. B.* **91**, 081402 (2015).

38. W. Zhao, S. Wang, S. Chen, Z. Zhang, K. Watanabe, T. Taniguchi, A. Zettl, F. Wang, Observation of hydrodynamic plasmons and energy waves in graphene. *Nature* **614**, 688–693 (2023).

39. D. G. Purdie, N. M. Pugno, T. Taniguchi, K. Watanabe, A. C. Ferrari, A. Lombardo, Cleaning interfaces in layered materials heterostructures. *Nat. Commun.* **9**, 5387 (2018).

40. L. Wang, I. Meric, P. Y. Huang, Q. Gao, Y. Gao, H. Tran, T. Taniguchi, K. Watanabe, L. M. Campos, D. A. Muller, J. Guo, P. Kim, J. Hone, K. L. Shepard, C. R. Dean, One-dimensional electrical contact to a two-dimensional material. *Science* **342**, 614–617 (2013).

41. A. M. Goossens, V. E. Calado, A. Barreiro, K. Watanabe, T. Taniguchi, L. M. K. Vandersypen, Mechanical cleaning of graphene. *Appl. Phys. Lett.* **100**, 1–4 (2012).

42. K. J. Tielrooij, N. C. H. Hesp, A. Principi, M. B. Lundeberg, E. A. A. Pogna, L. Banszerus, Z. Mics, M. Massicotte, P. Schmidt, D. Davydovskaya, D. G. Purdie, I. Goykhman, G. Soavi, A. Lombardo, K. Watanabe, T. Taniguchi, M. Bonn, D. Turchinovich, C. Stampfer, A. C. Ferrari, G. Cerullo, M. Polini,

F. H. L. Koppens, Out-of-plane heat transfer in van der Waals stacks through electron-hyperbolic phonon coupling. *Nat. Nanotechnol.* **13**, 41–46 (2018).

43. Y. Cai, L. Zhang, Q. Zeng, L. Cheng, Y. Xu, Infrared reflectance spectrum of BN calculated from first principles. *Solid State Commun.* **141**, 262–266 (2007).
